# Diagnosis of thyroid micronodules on ultrasound using a deep convolutional neural network

**DOI:** 10.1038/s41598-023-34459-3

**Published:** 2023-05-04

**Authors:** Miribi Rho, Sei Hyun Chun, Eunjung Lee, Hye Sun Lee, Jung Hyun Yoon, Vivian Youngjean Park, Kyunghwa Han, Jin Young Kwak

**Affiliations:** 1 Department of Radiology, Severance Hospital, Research Institute of Radiological Science, Yonsei University College of Medicine, Seoul, Korea; 2 School of Mathematics and Computing, Yonsei University, Seoul, Korea; 3 Biostatistics Collaboration Unit, Yonsei University College of Medicine, Seoul, Korea

**Keywords:** Computational biology and bioinformatics, Medical research

## Abstract

To assess the performance of deep convolutional neural network (CNN) to discriminate malignant and benign thyroid nodules < 10 mm in size and compare the diagnostic performance of CNN with those of radiologists. Computer-aided diagnosis was implemented with CNN and trained using ultrasound (US) images of 13,560 nodules ≥ 10 mm in size. Between March 2016 and February 2018, US images of nodules < 10 mm were retrospectively collected at the same institution. All nodules were confirmed as malignant or benign from aspirate cytology or surgical histology. Diagnostic performances of CNN and radiologists were assessed and compared for area under curve (AUC), sensitivity, specificity, accuracy, positive predictive value, and negative predictive value. Subgroup analyses were performed based on nodule size with a cut-off value of 5 mm. Categorization performances of CNN and radiologists were also compared. A total of 370 nodules from 362 consecutive patients were assessed. CNN showed higher negative predictive value (35.3% vs. 22.6%, P = 0.048) and AUC (0.66 vs. 0.57, P = 0.04) than radiologists. CNN also showed better categorization performance than radiologists. In the subgroup of nodules ≤ 5 mm, CNN showed higher AUC (0.63 vs. 0.51, P = 0.08) and specificity (68.2% vs. 9.1%, P < 0.001) than radiologists. Convolutional neural network trained with thyroid nodules ≥ 10 mm in size showed overall better diagnostic performance than radiologists in the diagnosis and categorization of thyroid nodules < 10 mm, especially in nodules ≤ 5 mm.

## Introduction

The detection of thyroid nodules has substantially increased with the widespread use of high-resolution ultrasound (US), resulting in a high prevalence of 19–67% for thyroid nodules in the general population^1,2^. Approximately 7–15% of detected thyroid nodules are thyroid cancers^3^. In thyroid micronodules (< 10 mm), fine-needle aspiration (FNA) remains controversial because papillary thyroid microcarcinomas, defined as tumors < 10 mm in size, have shown near-zero cancer-specific mortality^4^. As it is difficult to predict which thyroid microcarcinoma will progress with clinical significance, most guidelines simply state FNA as an available option, leaving the decision up to clinicians to decide based on clinical settings and patient preference^5–8^.

Multifocality and bilaterality in papillary thyroid carcinoma are common features with a reported frequency of 18–87%^9^, and are known risk factors of nodal metastasis, distant metastasis, and regional recurrence after initial therapy^10^. The American Thyroid Association guideline first recommends lobectomy for unifocal papillary thyroid microcarcinoma without extrathyroidal extension but also notes that the presence of a bilateral nodule can suggest the need for a bilateral thyroidectomy to address the possibility of bilaterality^5^. To note, the US features used to differentiate benign and malignant thyroid nodules are equally applied to both macronodules and micronodules)^11,12^. Considering that physicians’ visual analysis of micronodules on US, especially of nodules smaller than 5 mm, has shown high false-positive rates, the preoperative detection of micronodules may increase additional FNA^13,14^. Furthermore, given the high nondiagnostic rate of FNA, preoperative diagnosis is still a challenging task for micronodules^10,14^.

The convolutional neural network (CNN) is a deep learning model which enables high-performance visual recognition and classification after automatically learning representative features from a training set^15,16^. The characteristics of the training set are therefore critical to the performance of CNN. CNN-based methods have been investigated to differentiate malignant and benign thyroid nodules and showed non-inferior or comparable diagnostic performance to radiologists^17–25^. Most studies have been conducted on thyroid nodules over 10 mm, and only three included thyroid nodules larger than 5 mm, but their mean size was larger than 10 mm^20,24,25^. Three other investigations have shown validation results for nodules corresponding to the same size criteria with training sets made up of nodules larger than 10 mm^18,21^ or 5 mm^20^, while no other study has demonstrated nodule size criteria in both the training and validation of CNN^17,19,22–25^. To the best of our knowledge, no study has applied a CNN-based model to thyroid nodules beyond the size criteria of the training set. In this study, we investigated the diagnostic performances of a CNN that was previously trained with thyroid nodules ≥ 10 mm to discriminate malignant and benign thyroid nodules < 10 mm and compare its diagnostic performance with those of radiologists.

## Methods

The institutional review board of Severance Hospital (Seoul, South Korea) approved this retrospective study, with a waiver for informed consent (IRB number: 2020-3659-001). Signed informed consent for biopsy or surgical procedures was obtained preoperatively from all patients. All methods were performed in accordance with relevant guidelines and regulations.

### Patients

**Figure 1:**
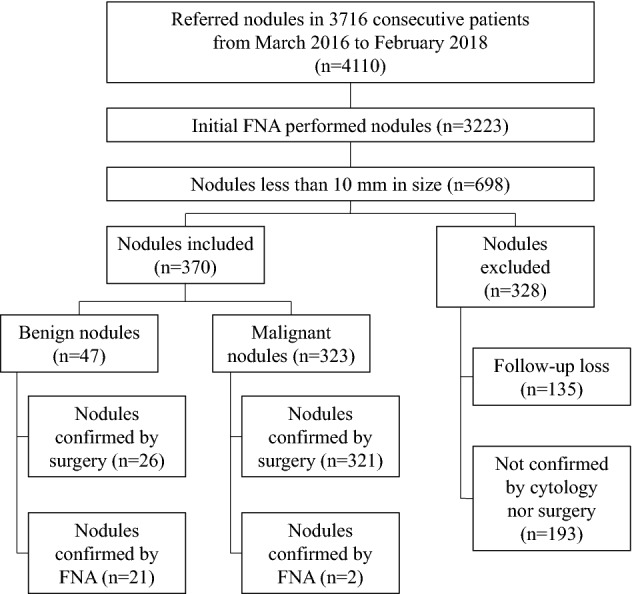
Flow chart of patient enrollment. A total of 370 nodules including 322 malignant nodules and 48 benign nodules were included in this study. *FNA* fine-needle aspiration.

This study was performed at a single tertiary referral center from March 2016 to February 2018, during which 4110 nodules in 3716 consecutive patients were consulted for US-guided FNA. The initial FNA was performed in 3323 nodules in 3240 patients, of which 698 nodules were < 10 mm in 683 patients. Our study included nodules < 10 mm if they (a) were cytologically confirmed as benign or malignant (Bethesda category II or VI) or (b) were confirmed as malignant on postsurgical histology. We excluded nodules that were not confirmed or lost to follow-up. Finally, a total of 370 thyroid nodules in 362 patients were included and analyzed (Fig. 1). Two thyroid nodules were included for 8 patients, among which 6 patients had both malignant nodules and 2 patients had one benign and one malignant nodule.

### US imaging

US examinations of both thyroid glands and neck areas were performed using a 5–12 MHz linear array transducer (*i*U22, Philips Healthcare, Amsterdam, Netherlands). Real-time US scans and subsequent US-FNA were performed by 12 radiologists with 1–20 years of experience in thyroid imaging.

Each radiologist who performed the US and US-FNA/core biopsy procedures interpreted each US scan of the thyroid nodules and recorded US features prospectively in our institutional database^26,27^. US features including composition, echogenicity, margin, calcifications, and shape were recorded using descriptors that have been used from June 2012 to the present in our institution^28^. Each thyroid nodule was categorized according to the Thyroid Imaging Reporting and Data System suggested by the Korean Society of Thyroid Radiology (KSThR TIRADS) using pre-recorded US features^7^.

### Image acquisition and CNN evaluation

An experienced radiologist with 20 years of experience dedicated to thyroid imaging who was blinded to clinical information and pathological results selected and retrieved a representative US image for each thyroid nodule from the PACS and stored it in JPEG format. For each image, a square ROI enclosing the entire targeted thyroid nodule was manually labeled using the Paint program of Windows 10 by the same radiologist who retrieved the images.

We used a computer-aided diagnosis (CAD) program to assess the malignancy risk of 370 thyroid nodules on US images. The performance of a CNN algorithm differs by data set, that is, it highly depends on the data used to train its network. There are many pre-trained models and a few of their test results (accuracy, sensitivity, and specificity of 370 test data sets) are reported in Supplemental Table S1. As ResNet101 shows one of the best performances with current US images, this paper focuses on analyzing the results from transfer learning using ResNet101. The pretrained CNN model ResNet101^29,30^ was fine-tuned with 13,560 US images of thyroid nodules ≥ 10 mm in size (further details on the CAD program are provided in the Supplemental Material)^21^. ResNet101 is a deep neural network that was originally trained with 1000 object classes, 1,281,167 training images, and 50,000 validation images. The basic algorithm of the residual net family (ResNet-18,34,50,101, and 152) has been previously introduced^29^ and the paper achieved state-of-the-art results in image classification by taking a standard feed-forward ConvNet and adding skip-connections that bypassed a few convolution layers at a time. Each bypass/shortcut produced a residual block from which the convolution layers predicted a residual further used in the block’s input tensor. ResNet101 consists of 347 layers capable of learning rich feature representations of images with an image input size of 224-by-224. For transfer learning, 13,560 US images composed of 7160 malignant and 6400 benign nodule images were used. To balance the number of data sets, we used the left–right mirroring augmentation of 760 randomly selected benign images so that a final total of 14,320 images were used in training. Since the fully connected layer and classification layer at the end of the original pretrained network were configured for 1000 classes, they were replaced with new layers adapted to the new data set (benign and malignant) with learning rates for weights and biases set to 10 each. In the fine-tuning process, the stochastic gradient descent with a momentum optimizer was used to train the network, the initial learning rate was set to 10-4, 10 epochs were conducted, and the mini-batch size was set to 50. The momentum of the stochastic gradient descent optimizer was set to 0.9 and the learning rate dropped by a factor of 0.5 every 4 epochs. The model was validated with internal data (95 benign, 539 malignant) and external data from three different hospitals (429 benign, 761 malignant).

Using the CAD program, we calculated the risks of malignancy as continuous values ranging from 0 to 100% (CAD value). We also categorized nodules by designating categories based on the CAD value (CNN TIRADS) according to the predicted probability from KSThR TIRADS. CNN TIRADS category 2 was assigned to nodules with a malignancy probability < 3%, category 3 for a probability < 15%, category 4 for a probability < 60% and category 5 for a probability ≥ 60%^7^.

### Statistical analysis

For the reference standard, histopathologic results from FNA or surgery were used to confirm the final diagnosis of each thyroid nodule. If there was a discrepancy between the two results, the reference standard was the histopathologic result from the surgical specimen.

Baseline patient characteristics and nodal US features were compared between malignant and benign nodules with the Student’s *t*-test and Pearson’s χ^2^-test at the patient level and the logistic regression analysis with the generalized estimating equation method for clustered data in a nodule-level comparison. Areas under the receiver operating characteristics curve (AUCs) with 95% CIs were obtained and the TIRADS category and CAD value of each thyroid nodule were divided as either positive or negative according to the Youden index. We compared the diagnostic performances of the TIRADS category and CNN by analyzing the sensitivity, specificity, accuracy, positive predictive value, and negative predictive value using logistic regression with the generalized estimating equation method. AUC values were compared with the Obuchowski algorithm for clustered data^31^. The same statistical analysis was performed for the subgroup analysis separately according to nodule size with a cut-off value of 5 mm.

We assessed the categorization performances of CNN TIRADS and KSThR TIRADS using the likelihood ratio χ^2^-test and the linear trend χ^2^-test for each categorization system to determine heterogeneity (small differences in risk of malignancy among nodules in the same category) and monotonicity of gradients (whether the risk of malignancy of nodules increases as the category increases), respectively^32,33^. We also used the Akaike information criterion, which is a widely used estimator for model selection. Smaller Akaike information criterion values indicate a more informative model in terms of goodness of fit^34^.

Statistical analysis was performed using statistical software (SAS version 9.4, SAS Institute, Cary, NC, USA) and the R Statistical Package (Version 4.0.2, Institute for Statistics and Mathematics, Vienna, Austria). Two-sided *P* values < 0.05 were considered to indicate statistical significance.

## Results

### Patients and nodules characteristics

A total of 370 nodules in 362 patients (mean ages, 46 ± 12 years; range 20–76 years) made up of 289 (79.8%; mean ages, 46 ± 12 years; range, 20–76 years) women and 73 (20.2%; mean ages, 45 ± 12 years; range, 26–73 years) men, were included in the final study population (Fig. 1). There were 347 (93.8%) nodules which were confirmed with surgery and 23 (6.2%) nodules which were confirmed with FNA. FNA was performed in the 370 nodules because of requests from physicians at outside clinics (n = 127), high suspicion nodules > 5 mm (n = 123)^7^, the need to determine surgical extent in patients with bilateral nodules (n = 83), patient request (n = 30) and cervical lymph node metastasis (n = 7).

**Figure 2:**
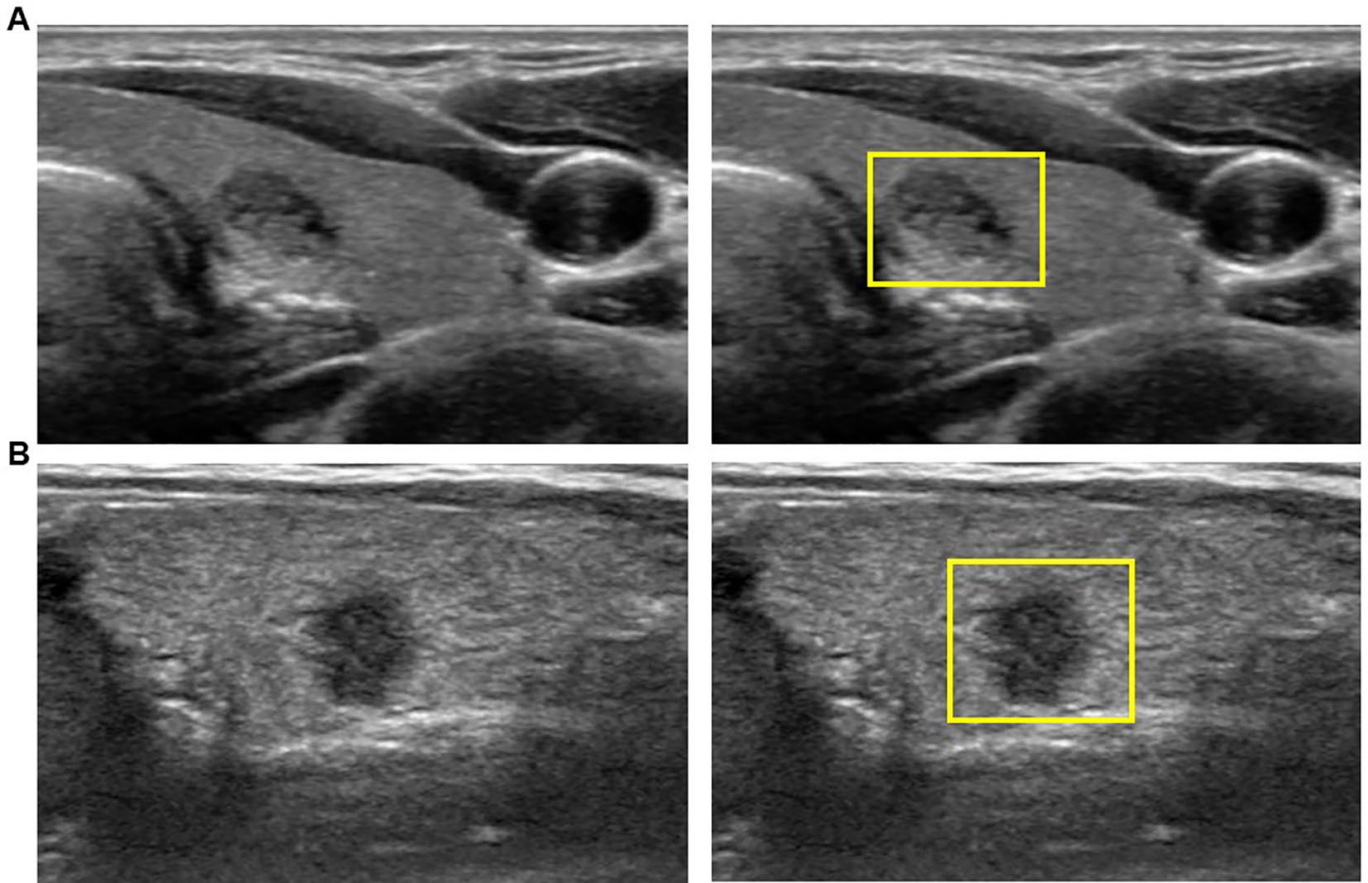
US image of 7 mm-sized thyroid nodules which were later diagnosed as malignant (papillary thyroid microcarcinoma) by surgical histopathology. The square ROI square enclosing the entire targeted thyroid nodule was labeled. (**A**) The nodule was categorized as KSThR TIRADS category 3 due to predominantly solid composition, mild hypoechogenicity, smooth margin, and parallel orientation without microcalcification. The malignancy probability calculated from CNN was 89.3%. (**B**) The nodule was categorized as KSThR TIRADS category 5 due to solid composition, hypoechogenicity, irregular margin, and non-parallel orientation. The malignancy probability calculated from CNN was 96.6%. *US* ultrasound, *KSThR* Korean Society of Thyroid Radiology, *TIRADS* Thyroid Imaging Reporting and Data System, *CNN* convolutional neural network.

**Figure 3:**
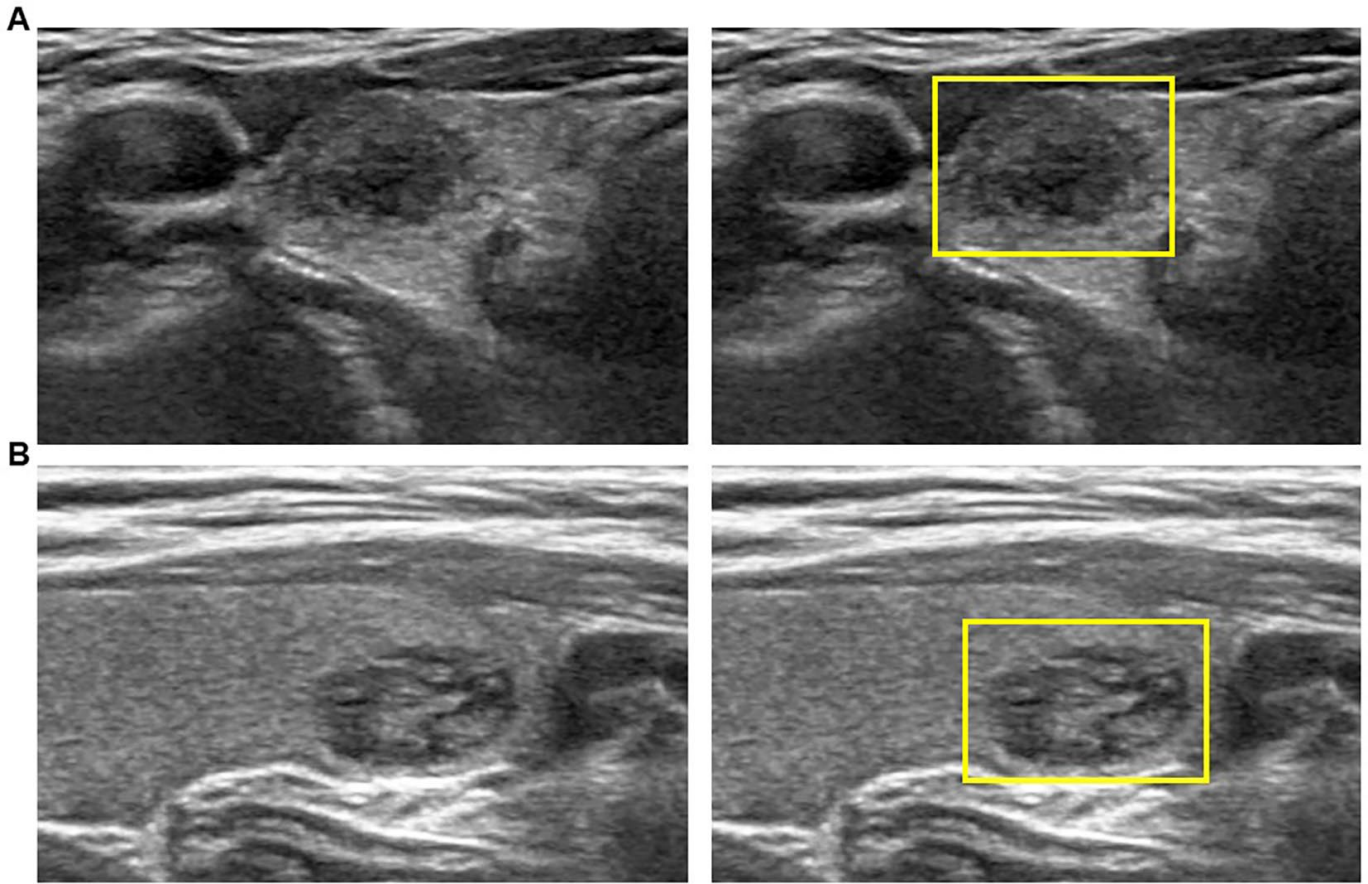
US image of 9 mm-sized thyroid nodules which were later diagnosed as Bethesda category II (benign follicular nodule) by FNA. The square ROI square enclosing the entire targeted thyroid nodule was labeled. (**A**) The nodule was categorized as KSThR TIRADS category 5 due to solid composition, mild hypoechogenicity, and microlobulated margin. The malignancy probability calculated from CNN was 5.8%. (**B**) The nodule was categorized as KSThR TIRADS category 3 due to predominantly solid composition, isoechogenicity, smooth margin, and parallel orientation. The malignancy probability calculated from CNN was 8.8%. *US* ultrasound, *FNA* fine-needle aspiration, *KSThR* Korean Society of Thyroid Radiology, *TIRADS* Thyroid Imaging Reporting and Data System, *CNN* convolutional neural network.

Among the 370 nodules, 323 nodules were confirmed as malignant and 47 nodules were confirmed as benign (Figs. 2 and 3). Of these malignant nodules, 322 nodules were confirmed as papillary thyroid carcinoma and 1 nodule as medullary thyroid carcinoma. The mean nodule size of the malignant and benign nodules was 5.3 ± 1.5 mm and 5.8 ± 2.2 mm, respectively (P = 0.14, Table 1). No significant difference was observed between the malignant and benign nodules for age (46.0 years vs. 45.9 years, P = 0.97) and female proportion (79.2% vs. 85.1%, P = 0.34).

**Table 1:** Patient demographics and nodal US features.

Characteristics	Malignant nodules	Benign nodules	Malignancy rate (%)^d^	*P*-value
No. of patients	317	47		
Age (years)^a^	46.0 ± 12.0	45.9 ± 13.0		0.97
Sex^b^				0.34
Female	251 (79.2%)	40 (85.1%)		
Male	66 (20.8%)	7 (14.9%)		
No. of nodules	323	47		
Nodule size (mm)^c^	5.3 ± 1.5	5.8 ± 2.2		0.14
KSThR TIRADS^c^				0.10
3	4 (1.2%)	3 (6.4%)	57.1 (18.7)	
4	37 (11.5%)	9 (19.2%)	80.4 (5.8)	
5	282 (87.3%)	35 (74.5%)	89 (1.8)	
CNN TIRADS^c^				< 0.001
2	1 (0.3%)	2 (4.3%)	33.3 (27.2)	
3	2 (0.6%)	3 (6.4%)	40 (21.9)	
4	36 (11.1%)	13 (27.7%)	73.5 (6.3)	
5	284 (87.9%)	29 (61.7%)	90.7 (1.6)	

All data except age and malignancy rate are numbers of patients or nodules, with percentages in parentheses.

*KSThR* Korean Society of Thyroid Radiology, *TIRADS* Thyroid Imaging Reporting and Data System, *CNN* convolutional neural network.

^a^Patient-level comparison using the Student’s *t* test for continuous variables. ^b^Patient-level comparison using Pearson’s χ^2^-test for categorical variables. ^c^Nodule-level comparison using logistic regression with the generalized estimating equation method. ^d^Standard errors are in parentheses.

### Comparison of diagnostic performance between the CNN and radiologists

The optimal cut-off points, set by the Youden index, were probability > 56.1% for CNN and KSThR TIRADS category 5 for radiologists. CNN showed higher AUC values than radiologists when diagnosing thyroid nodules (0.66 vs. 0.57, P = 0.04, Table 2). CNN also showed higher values for sensitivity (89.8% vs. 87.3%, P = 0.26), specificity (38.3% vs. 25.5%, P = 0.10), accuracy (83.2% vs. 79.5%, P = 0.08), positive predictive value (90.9% vs. 89.0%, P = 0.07) and negative predictive value (35.3% vs. 22.6%, P = 0.048).

**Table 2:** Diagnostic performance of the CNN and radiologists.

Performance measures^a^	CNN	Radiologists	P-value
True positive	290	282	
True negative	18	12	
False positive	29	35	
False negative	33	41	
Sensitivity	89.8 (86.5–93.1)	87.3 (83.7–90.9)	0.26
Specificity	38.3 (24.4–52.2)	25.5 (13.1–38)	0.10
Accuracy	83.2 (79.4–87.0)	79.5 (75.3–83.6)	0.08
PPV	90.9 (87.8–94.1)	89.0 (85.5–92.4)	0.07
NPV	35.3 (22.2–48.4)	22.6 (11.4–33.9)	0.048
AUC^b^	0.66 (0.57–0.75)	0.57 (0.50–0.63)	0.04

95% CIs are noted in parentheses.

*CNN* convolutional neural network, *PPV* positive predictive value, *NPV* negative predictive value, *AUC* area under the receiver operating characteristics curve.

^a^Each performance measure was compared using logistic regression with the generalized estimating equation method except for AUC. ^b^AUC was compared using the Obuchowski algorithm.

Among 370 nodules, 179 nodules were > 5 mm and 191 nodules were ≤ 5 mm. The characteristics of the patients and nodules are presented in the Supplemental Table S2. Age and portion of malignancy were not different between the subgroups divided by nodule size.

Cut-off values for the malignancy probability from CNN were redefined as > 55.8% for nodules > 5 mm and > 90.3% for nodules ≤ 5 mm. AUC values for diagnosing thyroid nodules did not differ between the CNN and radiologists in nodules > 5 mm (0.69 vs. 0.62, P = 0.25), while CNN showed higher AUC values than radiologists in nodules ≤ 5 mm with borderline significance (0.63 vs. 0.51, P = 0.08, Supplemental Table S3). In nodules ≤ 5 mm, CNN showed lower values for sensitivity (56.8% vs. 92.3%, P < 0.001) and accuracy (58.1% vs. 82.7%, P < 0.001) but higher values for specificity (68.2% vs. 9.1%, P < 0.001).

### Comparison of categorization performance between the CNN and radiologists

Among 323 malignant nodules, 4 (1.2%) nodules were category 3, 37 (11.5%) nodules were category 4 and 282 (87.3%) nodules were category 5 according to KSThR TIRADS (Table 1). Among 47 benign nodules, 3 (6.4%) nodules were category 3, 9 (19.2%) nodules were category 4 and 35 (74.5%) nodules were category 5. TIRADS categorization according to CNN showed higher values in the linear trend χ^2^-test (20.3 vs. 7.0) and likelihood ratio χ^2^-test (20.9 vs. 6.3) and lower Akaike information criterion values (264.8 vs. 279.4) than KSThR TIRADS assessed by radiologists, suggesting better categorization performance (Table 3).

**Table 3:** Comparison of categorization performance between the CNN and radiologists.

Test	Linear trend χ^2^ test^a^	LR χ^2^ test^a^	AIC^b^
CNN TIRADS	20.3	20.9	264.8
KSThR TIRADS	7.0	6.3	279.4

*CNN* convolutional neural network, *LR* likelihood ratio, *AIC* Akaike information criterion, *KSThR* Korean Society of Thyroid Radiology, *TIRADS* Thyroid Imaging Reporting and Data System.

^a^Higher values suggest better monotonicity of gradient and heterogeneity. ^b^Lower values suggest a more parsimonious model.

## Discussion

Our study demonstrated that when diagnosing thyroid nodules < 10 mm, CNN trained with thyroid nodules ≥ 10 mm showed better performance than radiologists. CNN also showed better performance than radiologists even in very tiny nodules ≤ 5 mm with borderline significance. In our study, we used a pretrained CNN which was fine-tuned with 13,560 images of thyroid nodules ≥ 10 mm and implemented it to smaller thyroid nodules < 10 mm.

CNN is an end-to-end model that automatically extracts features from digital images to enable pattern recognition, object detection, and classification. Since LeCun et al. proposed LeNet, the first CNN model in 1989, CNN has rapidly developed and various CNNs such as AlexNet or ResNet have been introduced^35^. The CNN-based diagnosis of thyroid nodules has shown comparable performance to experienced radiologists (Table 4). CNN has also shown significantly higher AUC values in recent studies using training sets with large numbers of nodules^19,21,22,25^. In addition, CNN has shown higher specificity than radiologists with similar levels of sensitivity (except in some studies using specific commercially available CAD)^19,21,25^.

**Table 4:** Comparison of diagnostic performance between CNN and radiologists in previous studies.

Author	Training set	Internal test set	External test set	Performances
Wang et al.^25^	5007 nodules	351 nodules, including 151 nodules < 1 cm	N/A	CNN showed significantly higher specificity and AUC than radiologists with comparable sensitivity
				In the subgroup of nodules < 1 cm, CNN also showed significantly higher specificity than radiologists
Li et al.^19^	42,952 patients	1118 patients	1574 patients	CNN showed significantly lower sensitivity and higher specificity than radiologists in both internal and external test sets
Buda et al.^22^	1278 nodules	99 nodules	N/A	CNN showed significantly higher specificity than inexperienced radiologists who did not use ACR TIRADS
				CNN showed similar AUC, sensitivity, and specificity to expert radiologists on the ACR TIRADS committee
Kim et al.^24^	Commercially available CAD	218 nodules ≥ 5 mm	N/A	CNN showed significantly lower specificity and AUC than radiologists with comparable sensitivity
Park et al.^20^	4919 nodules ≥ 5 mm	286 nodules ≥ 5 mm	N/A	No significant difference in diagnostic performance between the CNN and radiologists
Ko et al.^18^	439 nodules ≥ 1 cm and < 2 cm	150 nodules ≥ 1 cm and < 2 cm	N/A	No significant difference in diagnostic performance between the CNN and radiologists
Koh et al.^21^	13,560 nodules ≥ 1 cm	200 nodules ≥ 1 cm	600 nodules ≥ 1 cm	CNN showed significantly higher AUC in the internal test set, while no significant difference was shown in the external test sets
				CNN showed significantly lower sensitivity and higher specificity than radiologists in the internal test set and one of the four external test sets
Han et al.^23^	Commercially available CAD	454 nodules ≥ 1 cm	N/A	CNN showed significantly lower specificity and AUC than radiologists with comparable sensitivity

*CNN* convolutional neural network, *N/A* not applicable, *AUC* area under the receiver operating characteristics curve, *ACR* American College of Radiology, *TIRADS* Thyroid Imaging Reporting and Data System, *CAD* computer-aided diagnosis.

To the best of our knowledge, no studies have validated the diagnostic performance of CNN on a test set that has a size range different from that of the training set. Our study shows that CNN can diagnose nodules that are completely different in size from those in the training set with significantly better AUC and negative predictive value than experienced radiologists. This is largely consistent with previous studies^19,21^. Our study also shows that differences in specificity and AUC are more significant between the CNN and radiologists in very tiny nodules < 5 mm. Considering the high false-positive rate of FNA in very tiny nodules, we can expect CNN to reduce unnecessary FNA in clinical practice, especially in thyroid micronodules^13^.

In our study, the categorization of nodules on CAD values showed comparable or better stratification ability than KSThR TIRADS in terms of discriminatory ability and homogeneity^32–34^. Since the CNN TIRADS defines categories according to the predicted risk of malignancy suggested by KSThR TIRADS, CNN can help clinicians decide the next management step for patients such as whether to follow up or perform FNA under the existing TIRADS guideline. CNN has the potential to be used as a convenient tool that will reduce the burden of clinical triaging thyroid micronodules.

We acknowledge that there are several limitations to our study. First, the number of benign nodules is markedly lower than that of malignant nodules. Because micronodules only underwent FNA when they showed highly suspicious features, FNA-confirmed benign nodules were relatively rare, resulting in low negative predictive value values of both CNN and radiologists. Second, a majority of the malignant nodules were papillary thyroid carcinoma. Because follicular neoplasms or the follicular variant of papillary thyroid carcinoma exhibit distinctive US features, our result cannot be generalized to the diagnosis of other pathologic disease entities^36^. Third, radiologists manually selected key images and draw ROIs to be entered into the CNN, implying that the calculations made by CNN are inevitably operator-dependent. In a past study using support vector machine-based CAD, the diagnostic performance of computer-aided diagnosis for thyroid nodules varied significantly according to the experience of radiologists^37,38^. Further studies should be followed to evaluate the reproducibility of CNN.

## Conclusion

The deep convolutional neural network trained with thyroid nodules ≥ 10 mm showed overall better diagnostic and categorization performance than radiologists in thyroid nodules < 10 mm, especially those ≤ 5 mm.

## Supplementary Information

Supplementary Information.

## Data Availability

The raw data analyzed in the study are available from the corresponding author on reasonable request.

## Abbreviations

US: Ultrasound
FNA: Fine-needle aspiration
CNN: Convolutional neural network
TIRADS: Thyroid Imaging Reporting and Data System
KSThR: Korean Society of Thyroid Radiology
CAD: Computer-aided diagnosis
AUC: Area under the receiver characteristic curve
