# Supplementary material for: Diagnosis of thyroid micronodules on ultrasound using a deep convolutional neural network
Source: Sci Rep. 2023 May 4;13:7231. doi: 10.1038/s41598-023-34459-3 (PMC10160046; doi:10.1038/s41598-023-34459-3)
Supplement: Supplementary file 1 — Supplementary Information. [file 41598_2023_34459_MOESM1_ESM.docx]

**Supplemental Material**

**Computer-aided diagnosis (CAD) program using a pre-trained deep convolutional neural network (CNN)**

In a previous study, a CAD program equipped with pre-trained CNNs trained with ultrasound (US) images of 439 thyroid nodule images was introduced, demonstrating great potential for CNNs in thyroid US-related research in various applications. This incorporated a process called transfer learning. Typical deep learning algorithms require a huge amount of data to obtain reliable parameter values in layers. When this amount of data is not practically attainable, transfer learning is an alternative and one of the most widely used approaches in the deep learning field. Transfer learning uses model parameters (weights) from pre-trained models that were constructed for standard computer vision benchmark datasets. In this way, one can produce fast, accurate and reliable results with a relatively small amount of data. The CAD program in a past study utilized Vgg-nets that were developed by the Visual Geometry Group of Oxford University in 2014. Vgg-nets are composed of tens of layers and are considered to be “deep” CNNs when compared with previously built CNNs.

In order to improve diagnostic performance, we constructed a CAD program with ResNet101 and conduct transfer learning with 13,560 thyroid US images. The ResNet101 is a “ultra-deep (101 layers)” CNN affiliated with residual learning developed in 2015 by a Microsoft research lab. The ResNet101 was designed to optimize deep layers more easily by employing a ‘pre-conditioning’-concept and was found to greatly improve accuracy. There have been many variations and combinations with other nets for residual learning based models.

All nodules in the training set were 10mm or larger in size and were cytologically or surgically proven to be either malignant or benign (7,160 malignant and 6,400 benign nodules). Region of interests (ROIs) for the 13,560 thyroid US images were marked with colored boxes as shown in Fig. S1 by experienced radiologists who were blinded to the clinical information and the reference standard results. Then, only the locational information of the ROI box was applied to the original image to extract ROI-only cropped images so that the intensity of the US image was not disturbed by the colored ROI box. These cropped images were resized as 224x224 pixel images to fit the pre-trained ResNet101 and then a total of 13,560 images were used to finally train the CAD program. In this study, 370 US images were tested with this CAD program and the performance of the CNN was analyzed.


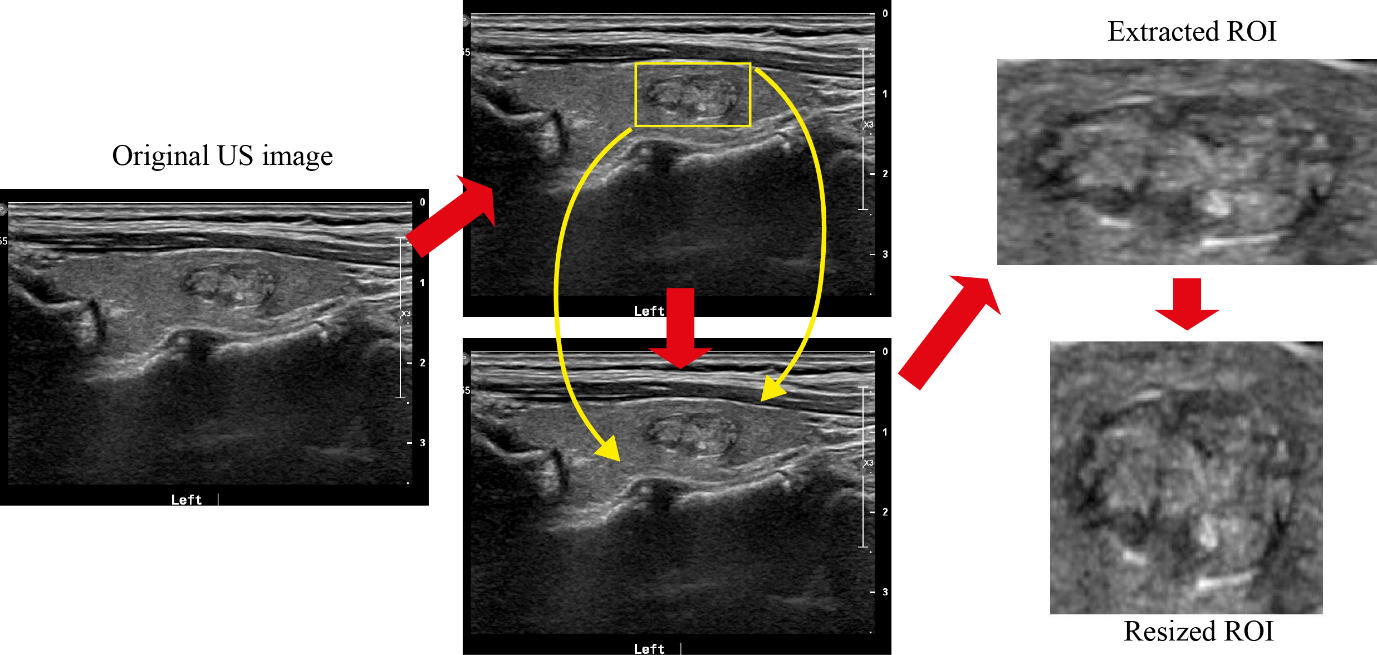
**Fig. S1.** ROI extracted from an US image and the resized ROI image for training CNN

ROI: Region of interest, US: ultrasound, CNN: convolutional neural network

**Supplemental Tables.**

**Table S1.** Performance comparison of various pre-trained models

| Pre-trained models | Accuracy (%) | Sensitivity (%) | Specificity (%) |
| --- | --- | --- | --- |
| GoogLeNet | 81.1 | 86.4 | 44.7 |
| ResNet18 | 75.1 | 80.8 | 36.2 |
| ResNet50 | 82.7 | 91.0 | 25.5 |
| ResNet101 | 83.2 | 89.8 | 38.3 |
| Inception-v3 | 76.2 | 80.5 | 46.8 |
| DenseNet-201 | 74.6 | 79.9 | 38.3 |
| DarkNet-19 | 83.0 | 92.6 | 17.0 |
| Xception | 85.7 | 97.5 | 0.04 |
| Inception-ResNet-v2 | 69.5 | 71.2 | 57.4 |
| NASNet-Large | 67.7 | 69.0 | 57.4 |

Test results (370 test data) of fine-tuned transfer learning using various pre-trained models. Training options are identical: the stochastic gradient descent with the momentum optimizer, the initial learning rate 10-4, 10 epochs(DenseNet-201, Darknet19, Xception: 5 epochs), and mini-batch size 50.

**Table S2.** Patient demographics and nodal US features according to nodule size

| Characteristics | Nodules > 5mm | Nodules ≤ 5mm | *P*-value |
| --- | --- | --- | --- |
| No. of patients^*^ | 177 | 188 |  |
| Age (years)^a^ | 47.5 ± 12.2 | 44.7 ± 11.9 | .03 |
| Sex^b^ |  |  | .53 |
| Female | 144 (81.4%) | 148 (78.7%) |  |
| Male | 33 (18.6%) | 40 (21.3%) |  |
| No. of nodules | 179 | 191 |  |
| Nodule size (mm)^c^ | 6.7 ± 1.0 | 4.1 ± 0.9 | <.001 |
| Nodular pathology ^c^ |  |  | .48 |
| Malignant | 154 (86%) | 169 (88.5%) |  |
| Benign | 25 (14%) | 22 (11.5%) |  |
| KSThR TIRADS^c^ |  |  | <.001 |
| 3 | 7 (3.9%) | 0 (0%) |  |
| 4 | 31 (17.3%) | 15 (7.9%) |  |
| 5 | 141 (78.8%) | 176 (92.2%) |  |
| CNN TIRADS^c^ |  |  | <.001 |
| 2 | 2 (1.1%) | 1 (0.5%) |  |
| 3 | 5 (2.8%) | 0 (0%) |  |
| 4 | 31 (17.3%) | 18 (9.4%) |  |
| 5 | 141 (78.8%) | 172 (90.1%) |  |

All data except age are numbers of patients or nodules, with the percentages in parentheses.

KSThR: Korean Society of Thyroid Radiology, TIRADS: Thyroid Imaging Reporting and Data System, CNN: convolutional neural network

^*^ 3 of these patients had nodules larger than 5mm and smaller than 5mm.

^a^ patient-level comparison using the Student’s *t*-test for continuous variables; ^b^ patient-level comparison using Pearson’s χ^2^-test for categorical variables; ^c^ nodule-level comparison using logistic regression with the generalized estimating equation method.

**Table S3.** Diagnostic performance according to nodule size

| Performance measures^a^ | CNN | Radiologists | p-value |
| --- | --- | --- | --- |
| Nodules > 5mm (n=179) | | | |
| True positive | 131 | 126 |  |
| True negative | 13 | 10 |  |
| False positive | 12 | 15 |  |
| False negative | 23 | 28 |  |
| Sensitivity | 85.1 (79.4-90.7) | 81.8 (75.7-87.9) | .37 |
| Specificity | 52.0 (32.4-71.6) | 40.0 (20.8-59.2) | .31 |
| Accuracy | 80.4 (74.6-86.3) | 76.0 (69.7-82.2) | .20 |
| PPV | 91.6 (87.1-96.2) | 89.4 (84.3-94.5) | .25 |
| NPV | 36.1 (20.4-51.8) | 26.3 (12.3-40.3) | .18 |
| AUC^b^ | 0.693 (0.566-0.819) | 0.615 (0.509-0.72) | .25 |
| Nodules ≤ 5mm (n=191) | | | |
| TP | 159 | 156 |  |
| TN | 5 | 2 |  |
| FP | 17 | 20 |  |
| FN | 10 | 13 |  |
| Sensitivity | 56.8 (49.3-64.3) | 92.3 (88.3-96.3) | <.001 |
| Specificity | 68.2 (48.7-87.6) | 9.1 (0-21.1) | <.001 |
| Accuracy | 58.1 (51.1-65.1) | 82.7 (77.4-88.1) | <.001 |
| PPV | 93.2 (88.3-98.1) | 88.6 (83.9-93.3) | .04 |
| NPV | 17.0 (9.2-24.9) | 13.3 (0-30.5) | .65 |
| AUC^b^ | 0.63(0.50-0.76) | 0.51(0.44-0.57) | .08 |

­

95% confidence intervals are noted in parentheses.

CNN: convolutional neural network, PPV: positive predictive value, NPV: negative predictive value, AUC: area under the receiver operating characteristics curve

^a^ Each performance measure was compared using logistic regression with the generalized estimating equation method except for AUC; ^b^ AUC was compared using the Obuchowski algorithm.
